# Microfabrication and optical properties of highly ordered silver nanostructures

**DOI:** 10.1186/1556-276X-7-292

**Published:** 2012-06-06

**Authors:** Hee-Ryoung Cha, Jaeseon Lee, Jae-Wook Lee, Jong-Man Kim, Jaebeom Lee, Jihye Gwak, Jae Ho Yun, Yangdo Kim, Dongyun Lee

**Affiliations:** 1 Department of Nano Fusion Technology, Pusan National University, Busan, 609-735, South Korea; 2 Solar Cells Research Center, Korea Institute of Energy Research, Daejeon, 305-343, South Korea; 3 School of Materials Science and Engineering, Pusan National University, Busan, 609-735, South Korea

**Keywords:** Physical vapor deposition, Raman spectroscopy, Nanostructured materials, Optical absorption, Surface-enhanced Raman scattering

## Abstract

Using thermal evaporation, we fabricated five uniform and regular arrays of Ag nanostructures with different shapes that were based on an anodized aluminum oxide template and analyzed their optical properties. Round-top-shaped structures are obtained readily, whereas to obtain needle-on-round-top-shaped and needle-shaped structures, control of the directionality of evaporation, pore size, length, temperature of the substrate, etc., was required. We then observed optical sensitivity of the nanostructures by using surface-enhanced Raman scattering, and we preliminarily investigated the dependency of Raman signal to the roughness and shape of the nanostructures.

## Background

Surface plasmon resonance (SPR) is a collective electron oscillation caused by electromagnetic (EM) radiation and its movement along the interface between metals and dielectric materials, which gives rise to a unique and extraordinary optical phenomenon, occurring especially in nanostructured noble metals (including particles and roughened thin films). This phenomenon is attracting a lot of interest from researchers because it enables the fabrication of powerful and highly sensitive detecting devices, i.e., optical, bio, and chemical sensing systems [1-5]. Silver and gold, with their sharp resonance peaks and high surface stabilities, are typical examples showing the SPR phenomenon [6]. The optical properties of metal spheres were described by Mie's theory in 1908 [7,8], and more recently, researchers have reported that SPR effects depend strongly on the geometry of the nanostructures because the EM wave formed around nanostructured noble metals is surface-confined and its energy could shift according to the nanostructure geometry [9-11]. Note that these descriptions have not been fully established [12,13]. Interestingly, several-thousand-fold enhancement of the optical sensitivity of silver fractal-like surfaces has been reported, and recently, this was also predicted theoretically [14-16]. Therefore, for the development of sensing devices employing SPR effects, fractal-like structures or highly ordered nanostructure arrays should be attractive candidates [17,18] because they might be able to guarantee the sensitivity of devices. Surface-enhanced Raman scattering (SERS) could be used to confirm the highly sensitive optical properties; this is the inelastic scattering of molecules on nanoscale metallic substrates [19] with high sensitivity and molecular specificity [9]. Raman enhancement is related to the characteristics of the nanoscale metallic substrates, such as the material, surface morphology, size, and aggregated state of the nanoparticles [19].

Recently, several methods for the fabrication of metallic nanostructures have been reported, including the engraving of periodic nanospheres, self-assembly of nanoparticles and nanowires, deposition of roughened thin films and nanoporous structures [19,20], and the creation of ordered nanostructures based on templates [21-24] by using the evaporation system [22,25] or lithography/etching technique [26-28]. However, the fabrication methods reported for highly ordered nanostructures are either difficult to obtain consistency or complicated and requiring expensive equipment. In this study, we would like to introduce an easy and reproducible method to fabricate highly ordered nanostructures by inexpensive equipment. We mainly focused on producing needle-like structures on substrate based on an anodized aluminum oxide (AAO) membrane template. These structures possess characteristics similar to those of the fractals with regard to shape and size [14]*.* We successfully fabricated various shapes of needle-like structures and examined them in order to understand how the nanostructure shapes affect their optical properties.

## Methods

AAO membranes (composed of AAO with a thin layer of Al metal) with various pore sizes and lengths were used as templates for the fabrication of highly ordered nanostructures of different shapes. Various types of AAO are commercially available, and we obtained three types with different lengths and pore sizes: 100 nm in length and 90 nm in diameter, 50 μm in length and 40 nm in diameter, and 50 μm in length and 80 nm in diameter (Nextron Co., Busan, Korea). The AAO membranes were cleaned in ethanol and deionized water before being loaded in a custom-made vacuum evaporation system (Georimtech Co., Daegu, Korea). High-purity silver (99.999% pure, Alfa Aesar, Ward Hill, MA, USA) was used as an evaporation source for deposition on the AAO template. All experiments were performed in a high-vacuum atmosphere of less than 1 × 10^−5^ Torr, and we used a specially designed holder for loading the AAO membrane. The AAO was then dissolved in 2 M NaOH solution at 40 °C for 24 h. The resulting Ag films, after removal of AAO, were cleaned with ethanol and then deionized water. The other side of the Ag films was attached to a Si wafer substrate for easy handling. The microstructures of the films were characterized by field-emission scanning electron microscopy (SEM) (Hitachi S-4700, Hitachi, Tokyo, Japan) and atomic force microscopy (Innova AFM, Veeco Instruments Inc., Plainview, NY, USA), which measured the roughness and curvature of the tips of the nanostructures. For investigation of the optical properties of the nanostructures, Raman spectroscopy (Lab Ram HR800, Horiba Jobin Yvon, Les Ulis, France) was used. In these measurements, we performed two ways to add dyes on the nanostructures: (1) each nanostructure was dipped in 10 μM Rhodamine 6 G (R6G) solution for 10 min, and (2) 50 μL of 10 μM R6G solution was dropped into each nanostructure for consistency of the amount of dyes on the nanostructures, and the R6G/nanostructures were analyzed with a 514.5-nm laser light (spec) with an angle of incidence of 0°.

## Results and discussions

**Figure 1:**
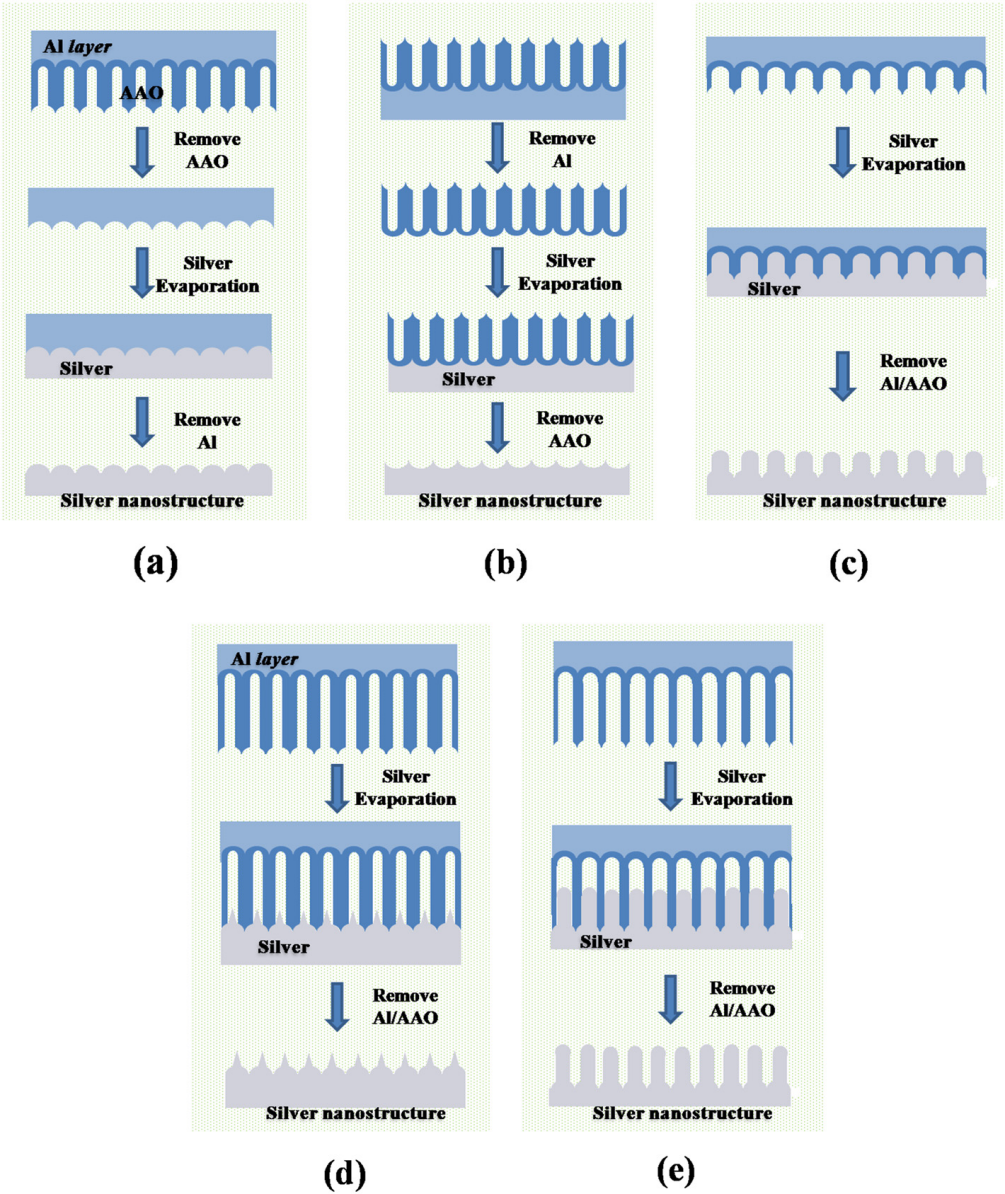
**Schematic diagram showing the fabrication of silver nanostructures using various types of AAO template. (a)** Round-top-hexagon, **(b)** sharp-wave-shape, **(c)** round-top-shape, **(d)** needle-on-round-top-shape, and **(e)** needle-shape structures.

**Figure 2:**
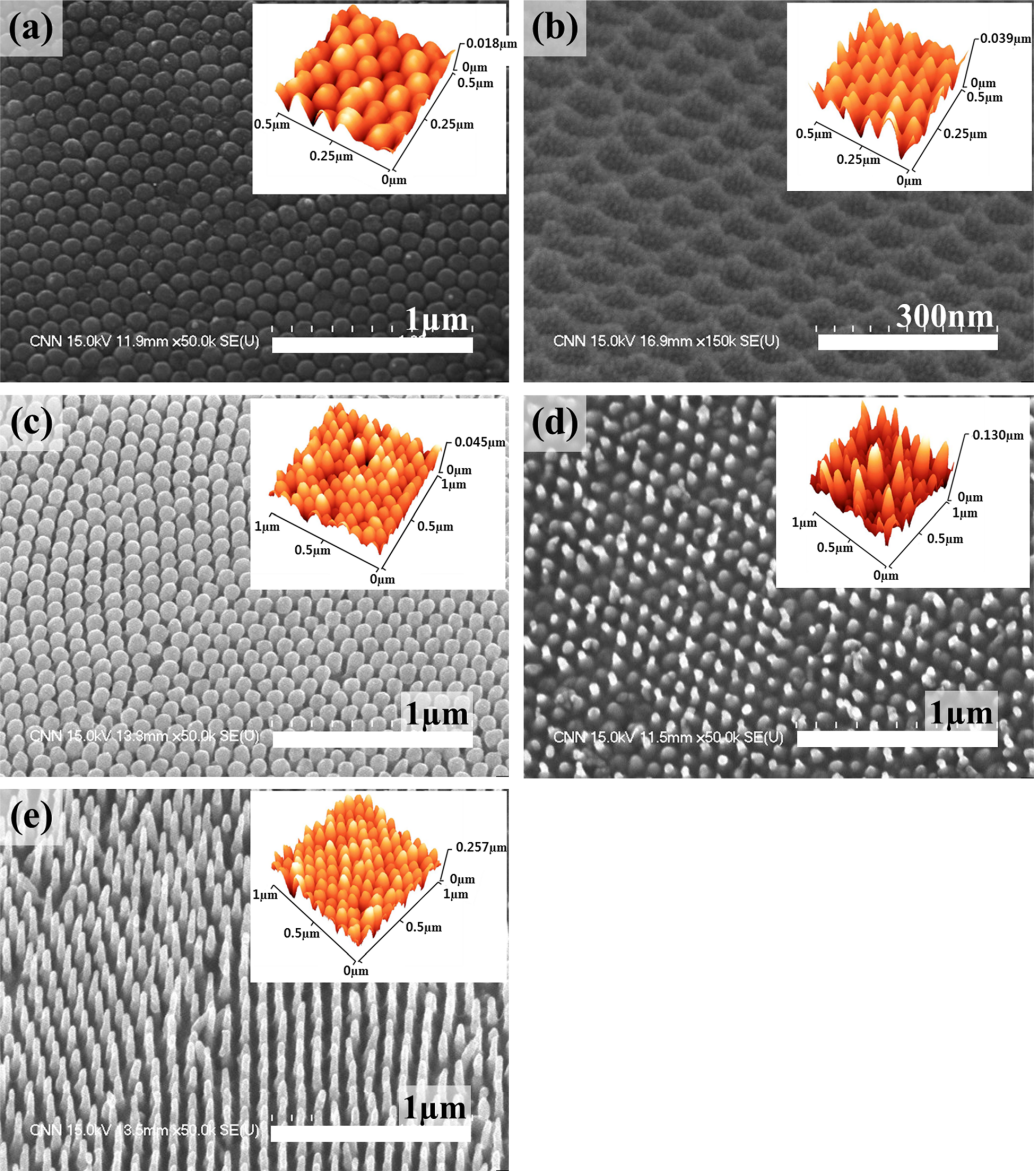
**SEM and AFM results.** 45° tilted view of SEM images and 30° tilted view of AFM images (insets) of each silver nanostructure: **(a)** RTH; **(b)** SWS; **(c)** RTS; **(d)** NRTS; and **(e)** NS.

With the purpose of this study mentioned in the ‘Background’ section, we demonstrated the fabrication technique of the five different-shaped nanostructures, which are shown in Figure 1, and gave a detailed clarification of these nanostructures from the SEM and AFM results in Figure 2. The detailed descriptions of the five different fabrication methods are as follows: (1) A round-top hexagonal (RTH) nanostructure was fabricated by evaporation of Ag onto a concaved surface of Al sheet followed by etched-away Al. The concaved Al sheet was prepared by removing AAO from the AAO/Al structure with a mixture of chromic acid (1.8 wt.%) and phosphoric (6 wt.%) acid solution at 60°C (Figure 1a). (2) A sharp-wave-shaped (SWS) nanostructure shown in Figure 1b could be formed by evaporation of Ag onto the bare AAO surface, which is the reverse structure of RTH because we removed Al instead of AAO from the AAO/Al membrane. AAO of the AAO/Al membrane was removed by a solution of CuCl_2_ (0.1 M) and HCl (20 vol.%) for 10 min at room temperature. (3) Another shape, named a round-top shape (RTS), can be formed simply by evaporation of Ag onto an AAO/Al membrane with a length of 100 nm and pore diameter of 90 nm (Figure 1c). In the formation of the RTS nanostructure, AAO with a relatively short length and large pore size provided a good conformal coverage that reflected its concaved bottom-shape. Because of the somewhat short length and wide opening of the AAO membrane, evaporated Ag molecules/atoms fill the AAO membranes before the evaporated Ag particles block the openings of the AAO. (4) Much longer AAO membranes with narrower pore sizes (50 μm long and 40 nm in diameter) were used to construct needle-on-round-top-shaped (NRTS) patterns, as shown in Figure 1d. Because of the smaller pore size and greater depth, the Ag particles evaporated could not penetrate deeply, and the capillary condensation effect gave rise to the NRTS structures. (5) Finally, longer and more needle-shaped (NS) nanostructures were successfully fabricated by using AAO membranes of the same length as that used to make the NRTS structures but twice wider, 80 nm in diameter. Detailed explanation for the formation of the specific nanostructures will be discussed later.

SEM and AFM micrographs of highly ordered silver nanostructures are shown in Figure 2. The silver films were characterized by AFM using tapping mode in air, which are presented in the insets of Figure 2. Special care was needed to get AFM images of the nanostructures, especially for the NRTS and NS structures, because of their extreme aspect ratio with a high density of the nanostructures. As shown in Figure 1, the RTH (Figure 2a) structures give a round-top shape since they reflect the bottom morphology of the AAO membrane whereas SWS (Figure 2b) structures, which also use the bottom morphology of the AAO membrane, show short and sharp wavy shapes because they reflect the outside form of the AAO membrane. The RTH and SWS structures are highly reproducible because they imitate the bottom shape of the AAO membrane, but the lengths of these structures are short. On the other hand, the RTS (Figure 2c), NRTS (Figure 2d), and NS (Figure 2e) structures show relatively longer and more needle-like shapes. These structures may not be obtained without special care; to improve conformal coverage, one has to carefully place the template, control the temperature of the template or the distance between the evaporating source and template to increase the directionality of evaporated species. The ‘capillary condensation effect’ mechanism suggested by Losic et al. [22] is an appropriate explanation for the formation of RTS, NRTS, and NS nanostructures. Although capillary condensation is the adopted terminology to depict phase transformation in nanoconfined surfaces [29], it is also a suitable mechanism for the fabrication of needle-like nanostructure arrays based upon nanosized templates. According to Losic et al., conical shapes of metal arrays can be realized easily by using a nanoscale template because capillary condensation is prevalent. Moreover, their suggestion could be developed to allow the fabrication of various shapes of nanoarrays by controlling the dimensions of the template, i.e., the pore size, length, and regularity, as confirmed in this study through the construction of RTS, NRTS, and NS nanostructures. In addition, without heating the template for more conformal coverage by diffusion, a similar effect could be obtained by improving the directionality of evaporated species that we obtained by reducing the evaporating distance between the source and the substrate (template). Although thermal evaporation has relatively good directionality, it depends strongly on the dimensions of the evaporation system. By using a specially designed holder for loading AAO membrane, which could control the distance from the evaporating source to the AAO template, we obtained a good conformal coverage. Note that an e-beam evaporation system would be the most appropriate equipment for this study, but it is excessively expensive compared to a thermal evaporation system.

**Figure 3:**
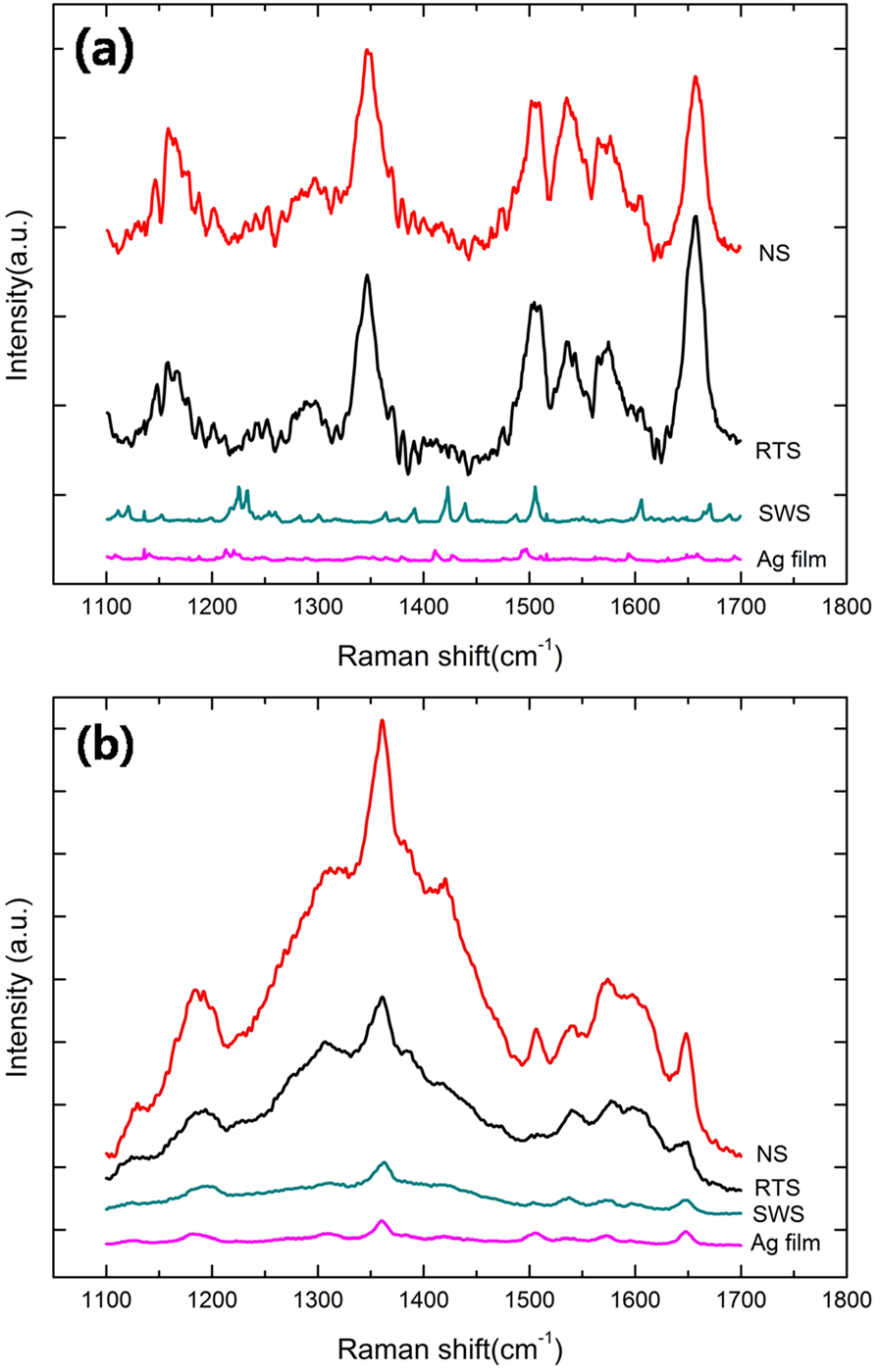
**SERS spectra of R6G adsorbed on fabricated needle-like Ag nanostructures (RTS, NS, and SWS structures).** A flat Ag thin film was also used for comparison. **(a)** Raman signal of the nanostructures dipped in 10 μM R6G solution for 10 min, **(b)** Raman signal of the nanostructures with 50 μL of 10 μM R6G solution dropped into them.

The roughness values and aspect ratios of the silver nanostructure arrays measured by AFM are presented in Table 1. The roughness values of the RTH, SWS, RTS, NRTS, and NS structures are 2.75, 5.64, 5.93, 17.04, and 39.3 nm, respectively. As expected, the order of aspect ratio of the nanoarrays is the same as that of the roughness of the structures because the roughness should be related to the length of the AAO membrane. In the case of RTH, RTS, NRTS, and NS, the nanostructures have well-arranged and dense hexagonal patterns of about 100 nm in diameter. However, unlike the other structures, the SWS structure is located at the angular point of the hexagonal patterns. From Figure 2a, it is clearly seen that the RTH structures show a dense hexagonal pattern with a diameter and height of about 100 and 10 nm, respectively. The SWS structure (the reverse structure of RTH) is about 20 nm in height. The RTS, NRTS, and NS structures are 100, 50, and 200 nm in height, respectively. Up to now, we demonstrated the simple/easy way to fabricate highly ordered and various shapes of nanostructures including needle-like nanorods. These ordered metallic nanostructures could be used for many applications, especially for optical, bio, and chemical sensing systems. We have performed a preliminary examination of these structures for optical applications measuring SERS effects presented in Figure 3.

**Table 1:** Roughness and aspect ratio of each silver nanostructure

	**RTH**	**SWS**	**RTS**	**NRTS**	**NS**
Roughness (nm)	2.75	5.64	5.93	17.04	39.3
Aspect ratio	0.1	0.3	1.1	1.3	2.6

NS, needle-shape; NRTS, needle-on-round-top-shape; RTH, round-top hexagon; RTS, round-top shape; SWS, sharp-wave-shape.

Figure 3 shows the SERS spectra of R6G molecules on the various silver nanostructures fabricated. In this study, we focused on three distinct structures (SWS, RTS, and NS) for evaluation of their optical properties by Raman spectroscopy. Figure 3a,b presents the spectra of nanostructures dipped in R6G and dropped R6G specimens, respectively. Almost all the distinctive peaks shown in Figure 3 correspond to the Raman lines for the R6G molecules. For comparison, we also used a silver thin film formed by evaporation, and it did not show any distinguishable enhanced Raman spectra. However, as shown in Figure 3, the RTS and NS structures showed a strong enhancement of the SERS spectra of the R6G molecules in both the dipped and dropped specimens. As known from literatures [19,30], the SERS effects depend strongly on the roughness of the metal nanostructure used as the substrate; we could expect that these three nanostructures would definitely enhance the SERS spectra. Interestingly, no significant SERS spectra enhancement was observed with the SWS nanostructures although they also present quite regular but rough surfaces. According to the results reported by Garcia-Vidal and Pendry [31], electromagnetic field enhancement effect is robustly dependent upon the geometry of the nanostructures, especially ratio of diameter, *d*, of nanorods and distance from tip to tip of nanorods, *R*. According to Garcia-Vidal and Pendry, they observed that plasmon could be trapped in between semicyliners, which locally create a huge ‘E’ field. In their conclusion, SERS is a very local phenomenon, and it could increase to as high as 10^7^ in the Raman signal. Their observation is widely adopted by many researchers [11,30,32], and electromagnetic field hotspot could also exist dependent upon the shape of nanostructures [27,28]. Therefore, RTS and NS structures are a more affordable geometry to enhance electromagnetic field that localized between needle-like rods. In SWS and other structures, the ratio of *d*/*R* may not demonstrate the increase Raman signal of the molecules. Furthermore, as shown in Figure 3, relative intensities of the Raman signal of NS and RTS are a little different depending on sample preparation methods, dipping or dropping. Presumably, this phenomenon could have resulted from the localization of E field hotspot depending on the geometry of the nanostructures. For example, E field will be strongly localized at the sharp tip of the structures [27,28]. An intensive study is now underway to depict how the tip shape of nanostructures will affect the SPR phenomenon.

## Conclusions

Consequently, we successfully fabricated five different silver nanostructures using a thermal evaporation system with an AAO membrane as the template. We demonstrated that using templates with different pore sizes enables the fabrication of different nanostructures. RTS structures were relatively readily obtained because of the shorter length to be filled in with silver by evaporation, whereas NRTS and NS structures required special care in terms of the directionality of evaporation, pore size, length, temperature of the substrate, and so on. In addition, we observed that SERS enhancement by Raman spectroscopy depends strongly on the roughness of the nanostructure. We also observed shape-dependent SERS enhancement. We are now examining how the shape and orientation of needle-like nanoarrays affect their optical properties. For this examination, high-resolution AFM imaging of individual silver structures and the corresponding cross-section analysis are underway. We expect that the tip curvature should be strongly dependent on the SPR effect.

## Abbreviations

SPR: surface plasmon resonance; EM: electromagnetic; SERS: surface-enhanced Raman scattering; AAO: anodized aluminum oxide; AFM: atomic force microscope; SEM: scanning electron microscopy.

## Competing interests

The authors declare that they have no competing interests.

## Authors' contributions

All the specimens used in this study and initial manuscript were prepared by HRC and JSL. JWL helped in the AFM scan. JMK, JBL, JHG, JHY, and YK put a valuable idea and useful discussion for this manuscript. All authors read and approved the final manuscript.

## References

[B1] Arai T Kumar PKR Rockstuhl C Awazu K Tominaga J An optical biosensor based on localized surface plasmon resonance of silver nanostructured films J Opt A: Pure Appl Opt 2007 9 699 703 10.1088/1464-4258/9/7/022

[B2] Haes AJ Zou S Schatz GC Van Duyne RP A nanoscale optical biosensor: the long range distance dependence of the localized surface plasmon resonance of noble metal nanoparticles J Phys Chem B 2004 108 109 116 10.1021/jp0361327

[B3] Homola J Yee SS Gauglitz G Surface plasmon resonance sensors: review Sens Actuator B-Chem 1999 54 3 15 10.1016/S0925-4005(98)00321-9

[B4] Wu J Lee S Reddy VR Manasreh MO Weaver BD Yakes MK Kunets Vas P Benamara M Salamo GJ Photoluminescence plasmonic enhancement in InAs quantum dots coupled to gold nanoparticles Mater Lett 2011 65 3605 3608 10.1016/j.matlet.2011.08.019

[B5] Wu J Makableh YFM Vasan R Manasreh MO Liang B Reyner CJ Huffaker DL Strong interband transitions in InAs quantum dots solar cell Appl Phys Lett 2012 100 051907 10.1063/1.3681360

[B6] Willets KA Van Duyne RP Localized surface plasmon resonance spectroscopy and sensing Annu Rev Phys Chem 2007 58 267 297 10.1146/annurev.physchem.58.032806.104607 17067281

[B7] Lal S Link S Halas NJ Nano-optics from sensing to waveguiding Nat Photonics 2007 1 641 648 10.1038/nphoton.2007.223

[B8] Aden AL Kerker M Scattering of electromagnetic waves from two concentric spheres J Appl Phys 1951 22 1242 1246 10.1063/1.1699834

[B9] Schwartzberg AM Zhang JZ Novel optical properties and emerging applications of metal nanostructures J Phys Chem C 2008 112 10323 10337 10.1021/jp801770w

[B10] Yoo HW Jung JM Lee S Jung HT The fabrication of highly ordered silver nanodot patterns by platinum assisted nanoimprint lithography Nanotechnology 2011 22 095304 10.1088/0957-4484/22/9/095304 21270483

[B11] Kelly KL Coronado E Zhao LL Schatz GC The optical properties of metal nanoparticles: the influence of size, shape, and dielectric environment J Phys Chem B 2003 107 668 677 10.1021/jp026731y

[B12] Zong RL Zhou J Li Q Du B Li B Fu M Qi XW Li LT Buddhudu S Synthesis and optical properties of silver nanowire arrays embedded in anodic alumina membrane J Phys Chem B 2004 108 16713 16716 10.1021/jp0474172

[B13] Feldheim DL Foss CA Metal Nanoparticles: Synthesis Characterization and Applications 2002 Marcel Dekker, Inc., New York

[B14] Aslan K Leonenko Z Lakowicz JR Geddes CD Fast and slow deposition of silver nanorods on planar surfaces: application to metal-enhanced fluorescence J Phys Chem B 2005 109 3157 3162 10.1021/jp045186t 16851335 PMC6848857

[B15] Lakowicz JR Radiative decay engineering: biophysical and biomedical applications Anal Biochem 2001 298 1 24 10.1006/abio.2001.5377 11673890 PMC6901132

[B16] Geddes Chris D Aslan K Gryczynski I Malicka J Lakowicz Joseph R Geddes Chris D, Lakowicz Joseph R Noble-Metal Surface For Metal-Enhanced Fluorescence Reviews in fluorescence 2004 Kluwer, New York 365

[B17] Li Z Kan C Cai W Tunable optical properties of nanostructured-gold/mesoporous-silica assembly Appl Phys Lett 2003 82 1392 1394 10.1063/1.1556563

[B18] Matsumoto F Ishikawa M Nishio K Masuda H Optical properties of long-range-ordered, high-density gold nanodot arrays prepared using anodic porous alumina Chem Lett 2005 34 508 509 10.1246/cl.2005.508

[B19] Qian L Yan X Fujita T Inoue A Chen M Surface enhanced Raman scattering of nanoporous gold: Smaller pore sizes stronger enhancements Appl Phys Lett 2007 90 153120 153120-3 10.1063/1.2722199

[B20] Kucheyev S Hayes J Biener J Huser T Talley C Hamza A Surface-enhanced Raman scattering on nanoporous Au Appl Phys Lett 2006 89 053102 053102-3 10.1063/1.2260828

[B21] Yang Y Tanemura M Huang Z Jiang D Li ZY Huang Y Kawamura G Yamaguchi K Nogami M Aligned gold nanoneedle arrays for surface-enhanced Raman scattering Nanotechnology 2010 21 325701 10.1088/0957-4484/21/32/325701 20639588

[B22] Losic D Shapter JG Mitchell JG Voelcker NH Fabrication of gold nanorod arrays by templating from porous alumina Nanotechnology 2005 16 2275 2281 10.1088/0957-4484/16/10/049 20818007

[B23] Padeste C Kossek S Lehmann HW Musil CR Gobrecht J Tiefenauer L Fabrication and characterization of nanostructured gold electrodes for electrochemical biosensors J Electrochem Soc 1996 143 3890 3895 10.1149/1.1837312

[B24] Lahav M Sehayek T Vaskevich A Rubinstein I Nanoparticle nanotubes Angew Chem Int Ed 2003 42 5576 5579 10.1002/anie.200352216 14639719

[B25] Ruan C Eres G Wang W Zhang Z Gu B Controlled fabrication of nanopillar arrays as active substrates for surface-enhanced raman spectroscopy Langmuir 2007 23 5757 5760 10.1021/la0636356 17425344

[B26] Wu W Hu M Ou FS Li Z Williams RS Cones fabricated by 3D nanoimprint lithography for highly sensitive surface enhanced Raman spectroscopy Nanotechnology 2010 21 255502 10.1088/0957-4484/21/25/255502 20508315

[B27] Kontio JM Husu H Simonen J Huttunen MJ Tommila J Pessa M Kauranen M Nanoimprint fabrication of gold nanocones with 10 nm tips for enhanced optical interactions Opt Lett 2009 34 1979 1981 10.1364/OL.34.001979 19571972

[B28] Kontio JM Simonen J Tommila J Pessa M Arrays of metallic nanocones fabricated by UV-nanoimprint lithography Microelectron Eng 2010 87 1711 1715 10.1016/j.mee.2009.08.015

[B29] Neimark AV Ravikovitch PI Vishnyakov A Bridging scales from molecular simulations to classical thermodynamics: density functional theory of capillary condensation in nanopores J Phys Condens Matter 2003 15 347 365 10.1088/0953-8984/15/3/303

[B30] Lang XY Chen LY Guan PF Fujita T Chen MW Geometric effect on surface enhanced Raman scattering of nanoporous gold: improving Raman scattering by tailoring ligament and nanopore ratios Appl Phys Lett 2009 94 213109 10.1063/1.3143628

[B31] Garcia-Vidal FJ Pendry JB Collective theory for surface enhanced Raman scattering Phys Rev Lett 1996 77 6 1163 1166 10.1103/PhysRevLett.77.1163 10063006

[B32] Khoury CG Vo-Dinh T Plasmonic nanowave substrates for SERS: fabrication and numerical analysis J Phys Chem C 2012 116 13 7534 7545 10.1021/jp2120669 PMC4022311 24839506

